# Aortogenic embolic stroke after sleeve pneumonectomy with median sternotomy for lung cancer: a case report

**DOI:** 10.1186/s13256-021-02796-4

**Published:** 2021-04-28

**Authors:** Hirotoshi Suzuki, Masafumi Noda, Tatsuaki Watanabe, Yasushi Matsuda, Yasushi Hoshikawa, Yoshinori Okada

**Affiliations:** 1 Department of Thoracic Surgery, Institute of Development, Aging and Cancer, Tohoku University, 4-1 Seiryo-machi, Aoba-ku, Sendai, Miyagi 980-8575 Japan; 2 Department of Thoracic Surgery, Fujita Health University Hospital, 1-98 Kutsugake-machi, Dengakugakubo, Toyoake, Aichi 470-1192 Japan

**Keywords:** Sleeve pneumonectomy with median sternotomy, Aortogenic embolic stroke, Diseased lung ventilation

## Abstract

**Background:**

The median sternotomy approach in sleeve pneumonectomy enables diseased lung ventilation in selected cases, which may reduce the difficulty in achieving anastomosis under intubation of the left main bronchus. However, with median sternotomy, the ascending aorta requires repeated mobilization to expose the operative field for anastomosis, which can cause an aortogenic embolic stroke.

**Case presentation:**

A 70-year-old Asian man presenting 6 months after developing hemoptysis was diagnosed with right upper lobe lung cancer (stage T4N0M0), invading the lower trachea and basal bronchus. Preoperative computed tomography revealed ascending aorta calcification. Right sleeve pneumonectomy was performed using median sternotomy with diseased lung ventilation. The ascending aorta was repeatedly mobilized to adequately expose the tracheobronchial bifurcation. Surgery was uneventful, but he did not recover complete consciousness even after termination of anesthesia. Mild paralysis of both upper extremities was observed. Head magnetic resonance imaging on postoperative day 1 revealed multiple small acute infarctions in the brain, possibly caused by mobilization of the aorta. He received anticoagulation therapy and rehabilitation and was discharged on postoperative day 30.

**Conclusion:**

The median sternotomy approach in sleeve pneumonectomy enables diseased lung ventilation. However, the possibility of aortogenic embolic stroke should be considered when calcification of the ascending aorta is observed on preoperative computed tomography.

## Background

Sleeve pneumonectomy is a surgical approach performed for extensive tumor resection involving the tracheobronchial angle, carina, or lower trachea [1–4]. Right sleeve pneumonectomy is usually performed via a right posterolateral thoracotomy in the fifth intercostal space. Median sternotomy offers several advantages, including less incisional pain, which results in less postoperative ventilatory restriction.

Brain infarction has not been reported as a postoperative complication following carinal resection with median sternotomy [2]. However, the patient in this report developed a brain infarction immediately after surgery, which was thought to be caused by repeated mobilization of the ascending aorta to expose the operative field for tracheobronchial anastomosis. We herein report the surgical course and postoperative findings of the patient.

## Case presentation

**Fig. 1:**
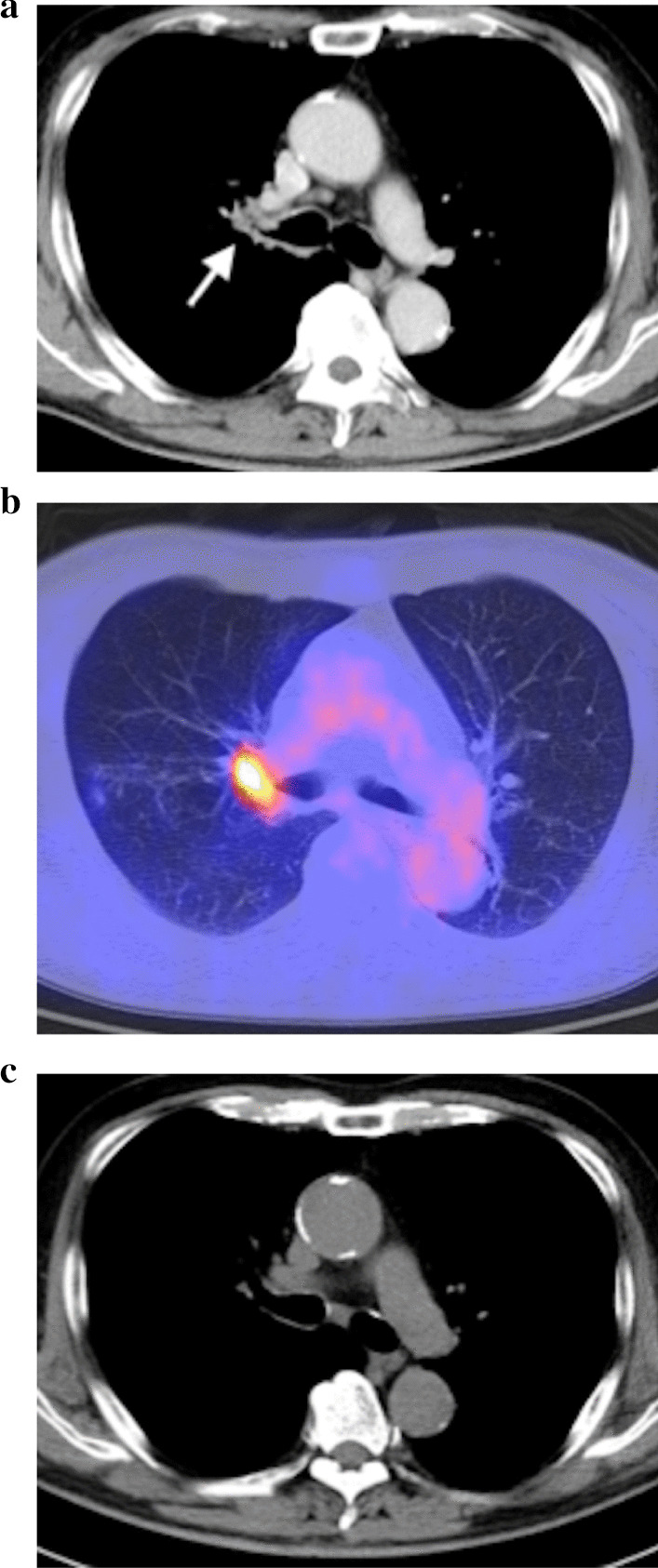
**a** Enhanced computed tomography showing a contrast effect in the tumor in the right pulmonary hilum and stenosis of the right main bronchus (arrow). **b** Positron emission tomography-computed tomography showing a tumor with a standardized uptake value of 8.5 in the pulmonary hilum. **c** Preoperative computed tomography showing semicircular calcification of the ascending aorta

A 70-year-old Asian man visited our hospital complaining of hemoptysis for 6 months. He was a pack-a-day smoker for 50 years and presented with hypertension. He was not taking any antiplatelet or anticoagulant agents. Chest X-ray revealed a mass in the right pulmonary hilum, and chest computed tomography (CT) showed a tumor in the pulmonary hilum with stenosis of the right upper lobe bronchus (Fig. 1a). Positron emission tomography-CT revealed a tumor in the pulmonary hilum with a standardized uptake value of 8.5 (Fig. 1b). Semicircular calcification of the ascending aorta was also noted (Fig. 1c). A bronchoscopic examination revealed redness and irregularity in the bronchial epithelium in an extensive area between the trachea and the lower lobe bronchus, with stenosis of the right upper lobe bronchus. Transbronchial biopsy of the lesion revealed a pathological diagnosis of squamous cell carcinoma. The results of biopsies from the lower trachea, one ring above the tracheobronchial angle and that of the B^6^/B^7–10^ spur, were also positive for squamous cell carcinoma. Endobronchial ultrasound-guided transbronchial needle aspiration showed no malignant cells in the subcarinal lymph nodes. The patient was able to tolerate right pneumonectomy, which was concluded from the results of a pulmonary function test; the patient’s forced expiratory volume in 1 second (FEV_1_) was 2850 ml (116.1% of the predicted value), and the diffusing capacity of the lungs for carbon monoxide (D_LCO_) was 15.80 ml/minute/mmHg (90.1% of the predicted value). Brain magnetic resonance imaging showed no metastasis or infarction. The clinical stage of the disease was T4N0M0, and right sleeve pneumonectomy was planned.

**Fig. 2:**
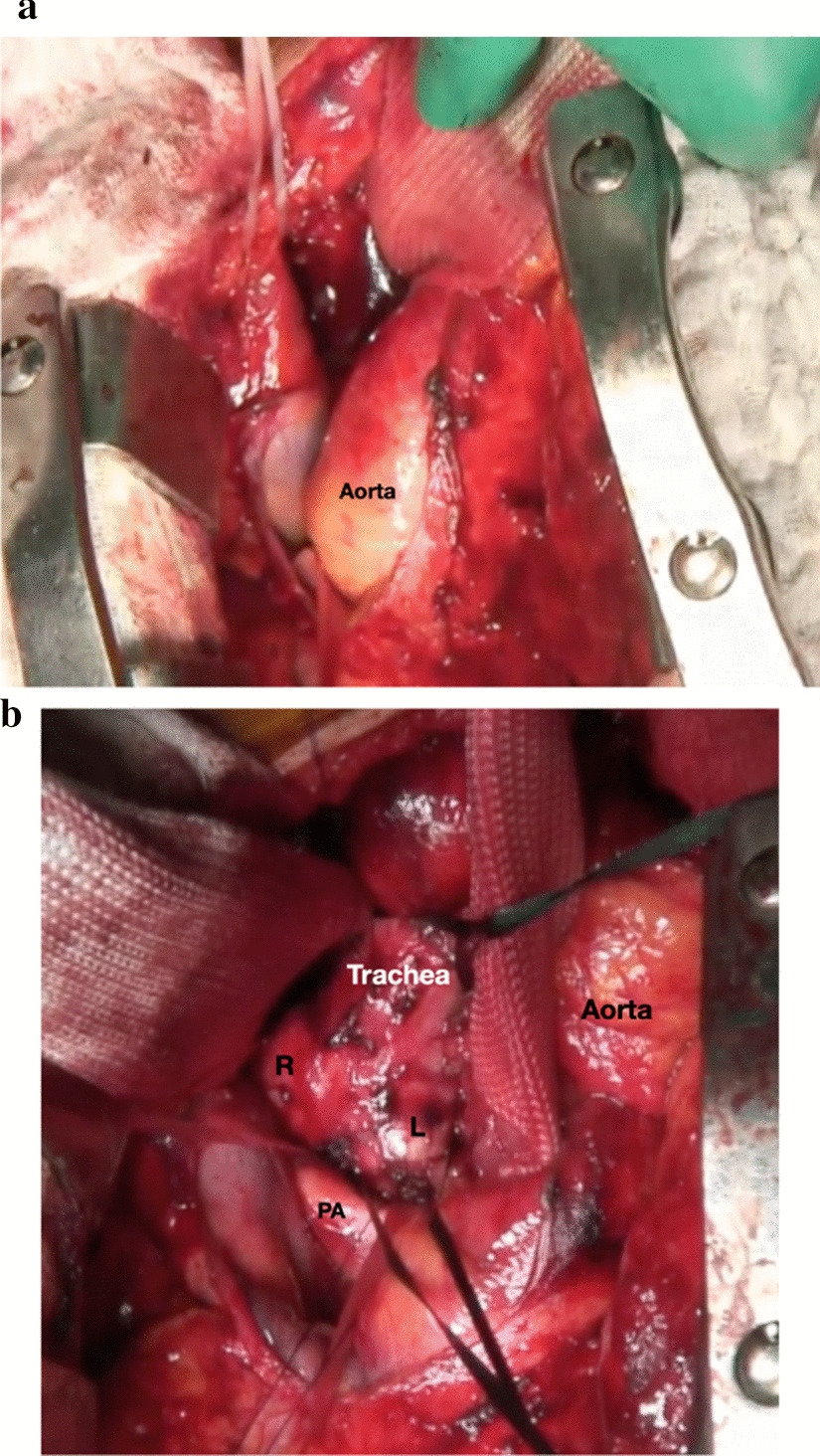
**a** Photograph before showing the operative field exposed by mobilizing the ascending aorta. **b** Photograph showing the operative field exposed by mobilizing the ascending aorta. *R* right main bronchus, *L* left main bronchus, *PA* pulmonary artery

Right sleeve pneumonectomy was performed using median sternotomy. During surgery, the ascending aorta was repeatedly mobilized to adequately expose the tracheobronchial bifurcation (Fig. 2a, b). Mediastinal lymph node dissection was performed, and the left main bronchus and the trachea were transected and anastomosed using 4-0 polydioxanone (PDS) under ventilation through the operative field. A running suture in the membranous portion was performed with ventilation of the diseased lung to better expose the operative field. The cartilaginous portion was anastomosed with interrupted sutures after the endotracheal double-lumen tube was forwarded into the left main bronchus. Following the anastomosis, the right main pulmonary artery and right superior and inferior veins were stapled, the right lung was extirpated, and right sleeve pneumonectomy was performed. After surgery, the patient was extubated and admitted to the intensive care unit; however, he was not fully conscious, and mild paralysis of both upper extremities was noted.

**Fig. 3:**
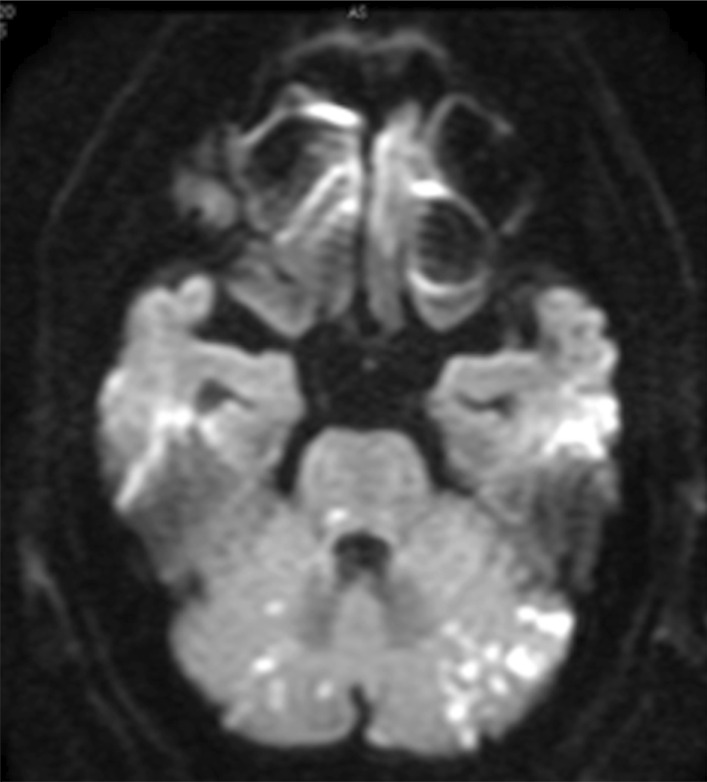
Magnetic resonance imaging showing multiple high-intensity areas in the brain, mainly cerebellum

Head magnetic resonance imaging was performed on postoperative day (POD) 1, which showed multiple high-intensity areas in the brain, mainly in the cerebellum (Fig. 3). Head magnetic resonance angiography (MRA) showed no significant change in the cerebral or vertebrobasilar arteries. No cardiac thrombus, malformation, or valvular disease was detected by echocardiography and contrast-enhanced CT, and the multiple embolic stroke was thought to be caused by repeated mobilization of the ascending aorta to expose the operative field for tracheobronchial anastomosis.

Heparinization therapy and administration of edaravone, a neuroprotective agent, were initiated. From POD 14, warfarin administration was initiated, and the amount of warfarin was adjusted to a prothrombin time-international normalized ratio (PT-INR) of 1.5–2.5. The patient received continuous anticoagulant therapy and rehabilitation and was eventually discharged on POD 30 without any sequelae. The patient is currently doing well without recurrence 5 years after surgery.

## Discussion

We present a case of multiple embolic strokes after right sleeve pneumonectomy with median sternotomy to treat lung cancer, which was associated with intraoperative repeated mobilization of the ascending aorta to expose the operative field. Sleeve pneumonectomy can be performed using either a posterolateral thoracotomy or median sternotomy approach, and the choice should be made after careful evaluation of the advantages and disadvantages of each approach. Although most surgeons prefer posterolateral thoracotomy, we believe that median sternotomy provides several merits (for example, less incisional pain). This approach also enables diseased lung ventilation in selected cases, which may reduce the difficulty in achieving anastomosis under intubation of the left main bronchus. However, with median sternotomy, the ascending aorta must be repeatedly mobilized to expose the operative field for anastomosis. Due to repeated mobilization of the aorta, the aortic plaque flows into the arteries of the brain, resulting in the development of multiple embolic strokes in the patient, mainly cerebellar infarction, possibly owing to surgery-associated embolism.

The prevalence of pulmonary embolization and thrombosis of the superior vena cava (SVC), a postoperative complication of carinal resection, is 1.29–1.7% [1, 2]. However, embolic stroke has not been reported as a postoperative complication following carinal resection using the median sternotomy approach [2]. Therefore, this is a rare case.

The magnetic resonance imaging characteristics of aortogenic embolic stroke are as follows: ≥ 3 lesions, lesions with a maximum diameter of < 30 mm, and vertebrobasilar system lesions [5]. All of these characteristics were observed in the present case, leading to the diagnosis of aortogenic embolic stroke. Shimada* et al*. reported an association between aortic arch calcification on chest radiography and aortogenic brain embolism [6]. As shown in Fig. 1c, the patient’s preoperative CT demonstrated semicircular calcification of the ascending aorta. It is suggested that atherosclerotic plaques ≥ 4 mm in thickness, ulcerated aortic plaques, and mobile aortic plaques in transesophageal echocardiography (TEE) are risk factors for ischemic stroke [7–9]. Considering the risk of embolic stroke by mobilization of the aorta, in cases of aortic calcification, TEE can help in choosing the approach for sleeve pneumonectomy.

Fortunately, the patient did not develop sequelae associated with multiple embolic stroke, and has been well for 5 years after surgery. However, it is important to consider the presence of aortic calcification when choosing the appropriate approach for sleeve pneumonectomy. To the best of our knowledge, this is the first reported case of multiple embolic stroke from the aorta, possibly associated with surgery after sleeve pneumonectomy. If a posterolateral thoracotomy approach in right sleeve pneumonectomy is selected in the presence of aortic calcification, the risk of embolic stroke can decrease.

## Conclusions

Aortogenic embolic stroke can occur after sleeve pneumonectomy with median sternotomy. The possibility of aortogenic embolic stroke caused by repeated mobilization of the aorta should be considered when calcification of the ascending aorta is observed on preoperative CT.

## Data Availability

Case report data and the patient’s consent form are available.

## Abbreviations

CT: Computed tomography
PT-INR: Prothrombin time-international normalized ratio
TEE: Transesophageal echocardiography
